# Discovery of and Interest in Health Apps Among Those With Mental Health Needs: Survey and Focus Group Study

**DOI:** 10.2196/10141

**Published:** 2018-06-11

**Authors:** Stephen M Schueller, Martha Neary, Kristen O'Loughlin, Elizabeth C Adkins

**Affiliations:** ^1^ Center for Behavioral Intervention Technologies Department of Preventive Medicine, Feinberg School of Medicine Northwestern University Chicago, IL United States

**Keywords:** mHealth, mental health, mobile apps, consumer preference, focus groups

## Abstract

**Background:**

A large number of health apps are available directly to consumers through app marketplaces. Little information is known, however, about how consumers search for these apps and which factors influence their uptake, adoption, and long-term use.

**Objective:**

The aim of this study was to understand what people look for when they search for health apps and the aspects and features of those apps that consumers find appealing.

**Methods:**

Participants were recruited from Northwestern University’s Center for Behavioral Intervention Technologies’ research registry of individuals with mental health needs. Most participants (n=811) completed a survey asking about their use and interest in health and mental health apps. Local participants were also invited to participate in focus groups. A total of 7 focus groups were conducted with 30 participants that collected more detailed information about their use and interest in health and mental health apps.

**Results:**

Survey participants commonly found health apps through social media (45.1%, 366/811), personal searches (42.7%, 346/811), or word of mouth (36.9%, 299/811), as opposed to professional sources such as medical providers (24.6%, 200/811). From the focus groups, common themes related to uptake and use of health apps included the importance of personal use before adoption, specific features that users found desirable, and trusted sources either developing or promoting the apps.

**Conclusions:**

As the number of mental health and health apps continue to increase, it is imperative to better understand the factors that impact people’s adoption and use of such technologies. Our findings indicated that a number of factors—ease of use, aesthetics, and individual experience—drove adoption and use and highlighted areas of focus for app developers and disseminators.

## Introduction

### Background

The number of publicly available mental health apps continues to expand at a breakneck pace. One estimate, as of 2017, proposed that nearly 325,000 health apps are available across the most common app stores (Google Play and iOS), a 25% increase from the previous year [1]. The exact number of mental health apps varies by definitions of what constitutes a *mental health* app, ranging from possibilities including general wellness apps to only disorder-specific mental health apps. Estimates suggest that about 7% of the market is focused on mental health [2], which would imply that 22,750 mental health apps exist. Indeed, more than 600 apps focus on depression, and 200 apps focus on suicide alone [3]. In light of this, various strategies have been proposed for how professionals and researchers might search for and understand these products [4]. However, there has been little investigation into how consumers with mental health needs search for and select health apps. In this study, first we conducted a survey of people with mental health needs and then conducted in-person focus groups to gain more detailed information. The goals of both the survey and focus groups were to better understand how people find health apps and information they use to guide their decision as to which apps to use.

Despite the wide availability of mental health apps, their impact on addressing the burden of mental health has been seriously lacking. This is largely because of the limited uptake and adoption of such tools both in routine care settings and by users in direct-to-consumer models. Furthermore, even those who do download a mental health app are unlikely to persist with that app over time. Two examples of publicly available apps with published information on their use are PTSD Coach [5-7] and IntelliCare [8]. In an evaluation of the uptake of PTSD Coach in the wild over 3 years, it was downloaded 153,834 times with 61.1% of people using the app within a day of installation, but only 41.6% using it after a month and 19.4% using it after 6 months [9]. In the case of IntelliCare, in the first year of its availability, 5210 people downloaded a total of 10,131 IntelliCare apps [8]. About half of the users continued to use the apps a day after installation, whereas a month after installation, rates of usage of each IntelliCare app ranged from 12.02% to 23.30% of the people who initially downloaded each respective app. It is worth noting that these 2 examples likely represent a best case for mental health apps, as these apps have empirical studies supporting their effectiveness [6,10]; were developed by well-respected government agencies and an academic medical school via government research funding; and are mentioned regularly in lay and professional audiences. Many mental health apps do not enjoy these benefits. About one-fourth of health apps downloaded are never opened, and 50% of health apps receive fewer than 500 downloads [11]. Although many might suggest that evidence supporting their efficacy or the development team should drive user adoption, the degree to which users value these factors in making choices to adopt a mental health app is unclear.

It has been suggested elsewhere that app adoption is a heuristic process that is guided by various informational cues [12]. Huang and Bashir [12] examined mental health apps intended to reduce anxiety and found that app ratings and reviews on app marketplaces correlated positively with the number of installs, whereas app price correlated negatively with the number of installs. Furthermore, app titles directly related to anxiety disorders or specific symptoms had lower rates of installation than apps with descriptions of the activities contained within the app such as mindfulness or journaling. A limitation of Huang and Bashir’s [12] study, however, was that it used observational data obtained directly from the app stores to understand what influences people’s adoption of mental health apps. Further information could be gleaned by directly asking consumers interested in mental health apps.

The most influential model related to the adoption of digital tools is the technology acceptance model (TAM) [13]. TAM proposes that the behavioral intention to use a technology precedes its use, which would result in uptake and adoption. TAM has been expanded to highlight the factors that influence behavioral intention including performance expectancy, effort expectancy, social influence, facilitating conditions, hedonic motivation, price, value, and habit in the unified theory of acceptance and use of technology (UTAUT) [14]. Although UTAUT is conceptually useful to highlight the constructs that impact people’s intentions and use, each of these constructs need to be operationalized with regards to specific technologies and populations. For example, cost has been noted to be an important determinant of health app adoption [15], but it also seems feasible that people might be willing to pay for apps that confer true health benefits. In the context of mental health, stigma might impact people’s willingness to talk about app use specifically or mental health generally that might impact social influence. As such, in this study, we aim to explore behaviors and perspectives as they relate to mental health apps in a population for which use of such apps would be relevant.

Finally, it is important to understand what information consumers are looking for to improve efforts to provide consumers with guidance for identifying and selecting mental health apps. Indeed, a recent report from a working group from the National Institute of Mental Health on opportunities and challenges of technology in clinical research concluded that “there is a need for rigorous evaluation and development of an evaluation structure of these apps” [16]. Several evaluation structures have been proposed either drawing from expert consensus such as the American Psychiatric Association’s App Evaluation Model [17] or from the synthesis of existing app rating structures such as the Enlight evaluation framework [18]. These structures share a multifaceted structure that considers several elements of the app such as research evidence, ease of use, therapeutic persuasiveness, privacy and security, and aesthetics. However, these models do not consider what factors are most important to consumers.

### Objectives

The field needs to better understand what consumers are looking for to build better products that incorporate those qualities and combine evidence-based practices that will result in effective and desirable mental health apps. Furthermore, understanding how people search for apps and what influences their decision to use an app may be helpful in presenting information about apps in persuasive ways to drive uptake and long-term use. This study addressed these issues through asking people about these questions using a survey and focus group methodology. Combining surveys with focus groups combines strengths of both approaches by collecting a large sample of respondents in surveys but eliciting more detailed and nuanced information in focus groups.

## Methods

### Study Design

We conducted a survey and focus groups to understand how people with mental health needs search for health apps and what information is valuable to consumers in making a decision as to the quality or desirability of particular apps. All participants were recruited from a research registry maintained by the Center for Behavioral Intervention Technologies (CBITs), which contains people who are willing to be contacted for future research opportunities. This research registry is framed as an opportunity to be involved in research on the use of technology to improve psychological well-being and improve general health with a particular focus on depression and anxiety. The survey was designed to take between 30 and 45 min to complete and could be completed remotely in exchange for entry into a lottery for a US $50 Amazon gift card. The focus groups lasted 90 min and are described in more detail below.

### Recruitment

An email blast was sent to members of the CBITs research registry, which contains 5100 members. Registry members living in Chicago were invited to complete a survey and a focus group but could complete either if they preferred. Registry members living outside Chicago were invited to complete the survey only. The survey link remained live for 8 weeks from a period of October through December 2017, at which point recruitment was suspended because of the high number of respondents. Inclusion criteria were ownership of a smartphone and being comfortable speaking in English. All recruitment and study procedures were approved by the Institutional Review Board of Northwestern University.

### Survey Sample

Of the 5100 registry members sent the survey link (both inside and outside of Chicago), 940 opened the survey, representing a response rate of 18.43% (940/5100). Moreover, 932 of these consented to participate and 811 completed the survey. Of the survey respondents, 79.5% were female (645/811), 18.3% were male (149/811), and 2.1% did not specify gender (17/811). The age range was 18 to 84 years (mean 36.1, SD 13.5). The majority of the sample was well educated, as outlined in Table 1. Although we did not ask about mental health symptoms in the context of our study, this information is collected when people enroll in the registry. The registry has elevated levels of symptoms of depression and anxiety with average scores of 14.3 (SD 5.4) on the Patient Health Questionnaire-8 and average scores of 12.2 (SD 5.4) on the Generalized Anxiety Disorder-7. Scores greater than 9 on the Patient Health Questionnaire and the Generalized Anxiety Disorder-7 are indicative of moderate depression or moderate anxiety and are recommended levels for referring people to treatment. We did ask whether survey respondents were receiving mental health treatments, and 57.9% (469/810) indicated they were, including 36.0% (292/810) receiving therapy and 51.1% (414/810) receiving medication. Thus, participants appeared to have mental health needs with many receiving mental health treatments.

These participants had experience with health apps generally. The average number of apps participants reported having on their phone was 54.14 (SD 50.89), with 3.12 (SD 4.35) of these or approximately 6% being health related (3.12/54.14). About one-third of participants (33.8%, 274/811) reported using a health app at least more days than not over the past week. A considerable minority reported they had not used a health app at all over the past week (28.8%, 234/811). Thus, although it seems that health app ownership was high, health app use was not. For mental health apps specifically, about one-third of the sample (33.9%, 275/811) indicated they had mental health apps on their phones. We discuss the results of the survey below.

### Focus Group Sample

In total, 163 eligible prospective participants expressed interest in a focus group, and a random selection were invited to a group. Seven focus groups were conducted with a total of 30 participants (23 females and 7 males) and an average of 4 participants per focus group (range of 3-6 participants in each group). Just under half (47%, 14/30) of the focus group sample had also completed the survey. Participants ranged in age from 21 to 72 years (mean 43.3, SD 14.3). The sample had varying levels of formal education, as outlined in Table 2. Participants reported high levels of confidence using a smartphone, demonstrated by level of agreement with the statement “I feel confident using a smartphone and downloading and using apps”; 27 participants strongly agreed, 2 agreed, and 1 neither agreed nor disagreed.

**Table 1:** Highest level of formal education completed by survey respondents.

Level of education	n (%)
Less than high school	5 (0.6)
High school graduate	38 (4.7)
Some college, no degree	178 (21.9)
Associate’s degree	62 (7.6)
Bachelor’s degree	306 (37.7)
Master’s degree	193 (23.8)
PhD	29 (3.6)
Total	811 (100.0)

**Table 2:** Highest level of formal education completed by focus group participants.

Level of education	n (%)
Less than high school	1 (3)
High school graduate	0 (0)
Some college, no degree	7 (3)
Associate’s degree	2 (7)
Bachelor’s degree	11 (37)
Master’s degree	6 (20)
PhD	3 (10)
Total	30 (100)

### Focus Group Procedures

The groups were held at Northwestern University’s CBITs office space. Participants received US $30 Amazon credit for their participation. Focus groups were semistructured, and facilitators (2 per group) took a flexible approach; questions were asked to guide the group through the relevant topics, whereas unanticipated ideas that emerged in the discussion were also pursued. The focus groups’ aim was to focus on mental health apps, and although we did discuss health apps generally, mental health topics roughly accounted for two-thirds of discussion with the groups. The full semistructured focus group guide is included in Multimedia Appendix 1. In brief, the focus groups were divided into 3 parts. The first was a discussion of mobile apps for health and participant’s experiences (both positive and negative) of using health apps. The second part was a discussion of mobile apps specifically for mental health. Again, participants were asked to share both positive and negative experiences using mental health apps as well as ideas on where to look for mental health apps and what information is important to them when choosing an app. The third and final part of the focus group focused specifically on PsyberGuide [19], a Web-based resource that identifies and reviews mental health apps. This study’s authors are responsible for the operation of and content on PsyberGuide with funding from One Mind, a nonprofit organization under which PsyberGuide is managed. Participants were guided through the website on a projector and gave feedback on content, design, and navigation. Learnings from the PsyberGuide portion of the interview were much more specific (eg, positive reactions to the PsyberGuide’s nonprofit status, increased desire for features to improve navigation and discoverability of apps, and a strong negative reaction to the word “product” as it conveyed commercial interests). These comments guided changes to the site content. We do not report on specific reactions to PsyberGuide further below, but PsyberGuide is available online.

## Results

### Survey Results

The most common source by which participants identified mental health apps was through social media (45.1%, 366/811) followed closely behind by their own searches (42.7%, 346/811). Common places people searched for apps were the app stores, Google searches, and Web forums such as Reddit. Although a considerable percentage did indicate that their medical providers were providing information about specific apps (24.6%, 200/811), even more participants indicated that a friend or family member helped them identify apps (36.9%, 299/811). As such, it seems like informal sources of information are relied on more than formal of sources of information in identifying mental health apps.

We also asked participants about the relative importance of a variety of features that might impact their adoption and sustained use of mental health apps. Participants responded on a 5-point Likert scale ranging from “not at all important” to “very important.” In Table 3, we display the number of participants who indicated that a feature was either “important” or “very important” to them, ordered in terms of rankings of most important to least important feature based on average responses. In general, the most important features related to the use of the app: is it easy to use and understandable? Issues related to privacy and data security (especially on the app side in terms of encryption compared with the user side in terms of a password) also appeared to be important.

We also explored what kept participants from downloading mental health apps to better understand barriers to uptake and adoption. The most common response was that participants were unsure how effective an app would be (31.4%, 255/811), although many fewer participants indicated that lack of research support contributed to this decision (6.6%, 54/811). Another highly endorsed barrier was about lack of knowledge regarding how to find an app or knowing which app to download (27.3%, 222/811). In general, other concerns were much lower including cost (13.7%, 111/811), lack of interest (11.1%, 90/811), privacy and data security (10.7%, 87/811), lack of time to use apps (6.6%, 54/811), lack of space on one’s device (6.0%, 49/811), and/or usability issues (5.0%, 41/811).

Finally, we asked participants about what they liked about current mental health apps. Findings from these questions were largely consistent with the patterns found across other questions. The most common response was related to ease of use (27.0%, 219/811), visual appeal (18.2%, 148/811), simple language (17.4%, 141/811), and content (14.4%, 117/811). Here, participants did not indicate fun (7.7%, 63/811) or name of the app (4.7%, 38/811) being particularly appealing aspects.

**Table 3:** Importance of features in mental health apps.

Feature	Responses, n		Total responses, n (%)
	Important	Very important	Important and very important
Content	324	412	736 (90.8)
Ease of use	321	406	727 (89.6)
Cost	222	420	642 (79.2)
Encryption	201	401	602 (74.2)
Interactive features	314	284	598 (73.7)
Customization	323	252	575 (70.9)
Privacy policy	195	377	572 (70.5)
Direct research evidence	271	293	564 (69.5)
Indirect research evidence	301	241	542 (66.8)
Simple language	277	215	492 (60.7)
User ratings	314	168	482 (59.4)
User reviews	293	183	476 (58.7)
Visual appeal	288	162	450 (55.5)
App description	268	161	429 (52.9)
Developer	208	199	407 (50.2)
Fun	238	159	397 (48.9)
Password protected	162	205	367 (45.2)
Graphics	214	124	338 (41.7)
Name	90	35	125 (15.4)

### Focus Group Results

#### Data Analysis

All sessions were audio-recorded and transcribed for coding. We conducted an inductive thematic analysis [20]. After sessions were transcribed, all transcripts were read with first memos and then open codes were created. After each transcript, codes were reviewed, which helped facilitate coding of subsequent transcripts. After all transcripts were memoed, coded, and initial themes identified, transcripts were read over again to identify which themes could be revised or combined. Themes were discussed among the study team, including the lead author who led the thematic analysis and the remaining authors who conducted the focus groups. We present the results of this process and provide quotes as specific examples of each theme within the results. We identify participants by number (eg, P1, P2, ...) and which focus group each participant was associated with (eg, FG1, FG2, ...).

#### Themes

We identified several themes related to people’s discovery and interest in health apps including the importance of personal use before adoption, desired features, and trusted sources. We discuss each of these themes along with related subthemes below.

#### Trusted Sources

One important source of information about which app to use was to lean on the recommendations of “trusted sources.” However, participants offered very different definitions of what a trusted source might be. Many participants identified “trusted sources” as people that they have an ongoing relationship with, be it a friend, colleague, or health care provider. For example, one participant stated:

If I’m gonna spend actual money or even stuff like that, I would want at the very minimum a recommendation from a friend, a person I trust, somebody saying, “I really like this one.”

P8, FG2

These participants indicated that such people might be more likely to make recommendations that reflect their preferences or needs or built off of something that had worked for them in the past.

However, participants also acknowledged the importance of professional or advocacy organizations in leading people toward effective products because of the perception that such groups would present less biased views or based recommendations on consensus and reviews of a variety of different apps:

I think it would be helpful, too, to have like the American Psychiatric Association or something, one of those, the licensure bodies or whatever—if they had official recommendations or backing, that would be nice to know.

P12, FG3

Finally, people generally indicated that connections to academic institutions or medical centers boost the credibility of apps. However, there were also some concerns about whether such organizations could produce apps that would provide the desired levels of usability and user experience. As one participant put it:

It’s a medical institution that made this app? It’s gonna be super shitty and really hard to deal with?

P7, FG2

Therefore, although such institutions may get a benefit from potential users in terms of expectations regarding effectiveness and safety, the trade-off in negative expectations toward usability and user experience means that these institutions need to ensure that they are comparable with similar apps created by other developers. In the end, however, although participants indicated such trusted sources were useful to inform initial uptake of apps, they seemed less important in supporting their long-term use.

#### Personal Use Guides Adoption

Despite the varied sources, participants reported they would rely on to make decisions regarding downloading apps, ultimately their own impressions and use tended to drive adoption. As such, in the searching phase, participants reported that other user reviews or screenshots were some of the most persuasive. One participant said:

The screenshots are probably going to be as important [as the developer], to kind of just see what the user interface is.

P19, FG5

Several participants commented on capabilities on both the Google Play and Apple iOS store to be able to see screenshots of apps and commented that these screenshots were extremely helpful to get a feel of user interface elements that would guide their decision to download the app.

However, a common theme for personal use is that many people do not simply pick one app and then use it. Instead, it was common for participants to report identifying multiple apps, downloading several, and then trying out those apps to be able to do direct head-to-head comparisons:

I have a tendency to go find many other apps of the same thing, and decide which one I like, to be honest with you.

P15, FG4

Participants noted that this was useful because with many of these apps, they were not sure which features they were looking for until they used it, and aspects about aesthetics, usability, or usefulness of particular features would become more apparent when it could be compared with other options.

Although cost was not a deterrent for participants, many participants did mention the need to preview the app before committing to pay for it. As P28, FG7 put it *“* free always wins*”* with the ability to view content before any purchases being a large factor in that decision. If there was a cost associated with an app, participants preferred in-app purchases or subscriptions that unlocked additional content compared with those that had even a small fee associated with it from the start:

So, if they don’t have the free trial and they want money, I’m not even gonna look at it. I’m not gonna pay for something before I’ve gotten the chance to see if it’s gonna work for me or not; free always wins.

P11, FG3

It is worth noting with regards to cost that participants did have thoughts about the value of apps with ongoing costs such as subscriptions. Although participants reported that they would pay *some* ongoing cost for an app they perceived as useful, many participants voiced some sort of limit to how much they would be willing to spend. For example, one participant mentioned one app that:

...they gave the option to pay $50.00 a year. And I did that, because I liked the idea of what they were trying to do, kind of create a social community of people.

P3, FG1

Other participants stated:

I wouldn’t spend $100.00 on any app for a year.

P13, FG3

...well, no, I’m not likely to buy a $60.00 a year app. Screw that. Never mind.

P7, FG2

Thus, although free may be a strong determinant of an initial decision to at least download and try an app, cost might figure differently when long-term use and benefit is considered.

#### Features

In general, participants wanted apps that were useful, easy to use, and aesthetically pleasing. Across participants, there were commonly reported desired features within apps including tracking, analytics (eg, reports and insights based on tracked data), data sharing, and notifications. Data sharing referred to opportunities to send and share data with others either directly, through social features or social media, or to other apps. In fact, participants saw apps’ ability to function for multiple uses or to connect to other data sources as related to usefulness and ease of use as it could reduce the burden for the user for data entry or increase meaningfulness of data through connections to other information. One of the most commonly discussed apps during the focus groups was *Clue*, a period and ovulation tracker. Many participants commented on *Clue* ’s ability to track a variety of symptoms related to one’s mental state such as focus, distraction, calm, and stress. This ability was useful to make connections between one’s cycle and mental health and to notice other patterns in one’s mental health more generally.

Usability was a major concern of participants that tended to differentiate those apps that would enjoy long-term use to those that would be quickly discarded. One subtheme within usability was the discoverability of different features. Many participants decried complicated multifeatured apps with “busy” home screens and the need to go through several screens. One participant said:

And for me, it’s just too overwhelming and too discombobulating. I just want to tap in and get the information that I need without clicking and searching for dear life.

P14, FG4

Another usability subtheme was the intuitiveness of apps, either through using paradigms or models that were similar to other commonly used apps or using language or visual elements that made the app quick to learn and use. The last subtheme in usability was bugs and technical difficulties. Many participants reported many apps associated with medical institutions have issues such as crashes, poor displays on their devices, or high demands on their phones memory. Usability was also strongly related to the other theme of personal use guiding adoption; participants reported that they were not willing to work through an app with significant usability concerns even if they could see it being beneficial.

Finally, participants preferred visually appealing apps, although the sentiment of P13, FG3 that “It has to be cute” was not universal among our participants, many commented on different aspects of aesthetics including color schemes, images, and the use of visual metaphors.

## Discussion

### Principal Findings

The large number of mental health apps means that consumers are faced with a considerable challenge to find any particular app. As consumer strategies for finding and selecting apps will likely bias downloads and use toward particular products, it is important to know how and why consumers make their selections. The results of our survey and focus group were largely consistent showing that, in general, content within apps (eg, aesthetics, features, and functionality) was the largest determinant to encourage people to download and use health apps. Although notions of credibility and issues of privacy and security were important, these aspects were often assumed to be present when “trusted sources” were involved in app development. There were also places where the results were potentially discrepant, which highlights some interesting areas for future work. For example, the survey data revealed one hesitation to adoption was uncertainty regarding the effectiveness of digital tools, but the focus group participants did not seem to think that research evidence was extremely compelling. It is possible that our survey responses reflect a broad question on whether digital tools for mental health could even reasonably be effective, especially with some concerns on the mental health impact of technologies such as smartphones and social media more broadly.

It is worth noting that people rely on relatively informal means of identifying apps, relying on Web searches, social media, and word of mouth. Consumer strategies are not wholly different from strategies recommended by Boudreaux et al [4] with the exception that consumers were unlikely to review the scientific literature and do not have professional connections to rely on. As such endorsements, such as that Apple made of *Calm* for the app of the year [21], might have a strong impact on people’s uptake and use of such products. However, it is worth noting that such endorsements are not based on research evidence and as such do not necessarily mean that such products are the most beneficial. Future work should help promote standards related to the promotion of health apps to ensure that effective tools make their ways into the hands of consumers. Furthermore, our focus groups identified “trusted sources” as a strong influence of people’s decision to use tools, but the survey results participants rarely received information about apps from professional sources such as providers. It is useful to consider ways to better involve providers in conversations around mobile apps. This might involve learning from providers what types of tools they would be interested in recommending or training providers as to what tools are safe, efficacious, and evidence-based. Indeed, some evidence suggests that people are likely to follow-up on their providers’ recommendations, especially in mental health [11], and aligning consumers’ needs and preferences with providers’ knowledge and recommendations will likely be a key piece of adoption in practice.

In terms of adoption of apps, a considerable amount of discussion in the focus groups revolved around early use and especially the first-time user experience (or what is referred to as the “FTUX”). Some apps mitigated this concern by relegating more advanced features or content to premium versions, which had the added benefit of a revenue stream for the app. Aspects that users were especially mindful of in the early experience were the usability, aesthetics, and visual interface elements. It is worth noting that for many mental health apps, it is unlikely that a single use would lead to the proposed benefit (eg, reduced depression or anxiety), as addressing many mental health concerns requires sustained behavior change. In light of this, developers should consider how to give users appropriate previews of the apps that not only give a sense of the look and feel of the app and the functionality of its feature but also whether or not the app is likely to lead to the proposed benefit. A stronger focus on the proximal outcomes of success early in the app journey might be critical for setting appropriate expectations and promoting long-term use.

Relatedly, it is worth noting that although users were strongly motivated by information about whether or not an app would help them, this information was not necessarily research evidence. As such, even though researchers have noted that many apps are not based on evidence-based principles [22] and that most have not undergo rigorous research evaluation [23], this is unlikely to impact consumer behavior. Consumers were much more likely to be interested in a variety of sources of information about expected benefit, including user reviews, anecdotes from friends or family members, or doctor recommendations. Therefore, it might be useful to conduct more structured evaluations leveraging similar logic (eg, n-of-1 designs, multiple baselines) to expand the type of evidence that consumers are looking for. Currently, several efforts to identify and review mental health apps exist (for a review, see the paper by Neary and Schueller [24]); it is possible that such reviews or certification from such sources could represent a “trusted source” of information, but we did not evaluate this directly in our survey or focus groups.

An interesting consideration is if one app could accomplish everything users need in the mental health space. Several participants commented positively on interoperability that allowed connection to other apps or leveraged features such as Apple’s Health or Google Fit. However, the downside to having an app accomplish multiple features is increased complexity that might reduce usability. As 2 examples of how this could be addressed, we can look at the Department of Defense apps (eg, PTSD Coach mentioned earlier) or IntelliCare [10]. The Department of Defense has produced several apps, many of which have similar interface elements or features. IntelliCare, on the other hand, has several apps that are interconnected through a hub app. The example of Clue provides another potential alternative in which mental health features are integrated into another health app. It could be that participants talked about health and mental health in this way because we asked questions specifically about health apps and mental health apps. However, it is worth noting that several common health apps, such as apps for physical activity and diet, might have connections to mental health, given the research showing linkages between these areas [25-27]. Apps for sleep may also have an important role to play in mental health, given that sleep is a common symptom of many mental health issues [28,29] and a target of several mental health interventions in both traditional [30] and digital formats [31]. Indeed, participants in our focus groups often cycled back to references to health apps while discussing mental health apps to find examples, make comparisons, or discuss commonalities. Not surprisingly, people talked about health and mental health apps similarly; the distinction between mental health apps and health apps might be more salient to mental health researchers and practitioners than to consumers. Going a step further, it could be that the wave of the future is neither health nor mental health apps, but more mental health app features integrated into commonly used apps like our calendars, conversational agents, or messaging platforms or apps focused on health more broadly. Integrating mental health in the operating system or basic applications would reduce the need to seek and find apps but would concentrate power in the hands of fewer developers who would then have a stronger influence on our health and mental health.

### Limitations

These findings, however, were not without their limitations, which are worth acknowledging to ensure that conclusions are accurately represented. First, participants in our study came from an established research registry and might not be representative of the more general population. Individuals willing to enroll in a research registry may be different from participants in other research studies because of their interest to participate in multiple research studies and willingness to be recontacted. The response rate of 18% was somewhat below average for Web-based surveys [32]; however, we did suspend recruitment after 8 weeks of data collection, which might have artificially reduced total participation. Still, our respondents might be biased toward those who are likely to respond more quickly, but it is unclear how such a characteristic might influence the results. Our respondents did tend to be well-educated and female; however, this also mirrors the characteristics of people who tend to enroll in research studies [33] and use technology-based mental health tools more broadly [34]. Furthermore, this registry, in particular, was connected with a group focused on research in digital mental health and as such participants may have more knowledge of digital mental health apps than other people. Nevertheless, given that apps are not currently widely recommended by health professionals, it is likely that interest drives adoption and this population might be representative of likely users. Another limitation is that it is unclear if when people were responding to survey questions, they were being descriptive (ie, how they would characterize the apps they use now) or prescriptive (ie, what they would like to see in apps that they would download and use). Therefore, we cannot conclude if answers relate to the current state of health and mental health apps or what consumers would be interested in seeing in future health and mental health apps.

### Conclusions

Mental health apps are a rapidly growing area with little indication that the speed of development will slow down. Although regulatory developments like approval from the United States Federal Drug Administration might impact their adoption, advances in this area are still too new to fully understand their long-term impact for both the marketplace and consumers. As such, better understanding factors that drive people’s decisions to download and use apps is an important step toward sustainable and impactful benefits from such technologies. Our findings highlighted a number of factors—ease of use, aesthetics, and individual experience—and also indicated that evidence-base and usefulness are not equivalent in the eyes of consumers. These findings can inform aspects of the design and dissemination of such products and hopefully impact efforts to ensure consumers get trusted and effective products.

## Appendix

Multimedia Appendix 1 Focus group scheduling, including semistructured interview.

## Abbreviations

CBITs: Center for Behavioral Intervention Technologies
TAM: technology acceptance model
UTAUT: unified theory of acceptance and use of technology
