# Supplementary material for: Discovery of and Interest in Health Apps Among Those With Mental Health Needs: Survey and Focus Group Study
Source: J Med Internet Res. 2018 Jun 11;20(6):e10141. doi: 10.2196/10141 (PMC6018235; doi:10.2196/10141)
Supplement: Multimedia Appendix 1 [file jmir_v20i6e10141_app1.pdf]

Focus Group Schedule

Welcome

I'm [interviewer name]. As you sit down please take some time to read through the consent form in front of you before you sign it. Please let me know if you have any questions or if you would like me to read through the consent form with you. We'll go through the main points in a few minutes when everyone is settled.

Background

- Thank you all so much for joining us today. We really appreciate you giving up your time to come in to talk to us.
- As you will read in the consent form, the purpose of this focus group is to learn about what users think about smartphone apps for health and mental health.
    - As technology continues to develop, we have more and more possibilities to use technology and our phones to help with health management. We want to know about your opinions of apps and your experiences using apps.
- Everything you say in this group will be audio recorded, so that we can go back later and listen to it. We are aiming to have 5 or 6 focus groups so when we are done, we want to listen to all of the data and see what the common themes or topics are.
- When we publish papers using this data, you will not be named or identified in any way.
- You are all here because you can help us understand what users want from health apps, and we really value your opinions so we invite you to join in the discussion as much as possible. However, you can contribute as much or as little as you like and if there are any questions you would prefer not to answer, that's ok.
- As we will be discussing health & mental health, if anyone discloses any personal or sensitive information we just ask that you respect their privacy and don't repeat that outside of this room.
- At the end of the focus group, you will get an $30 Amazon Credit and we will reimburse your transport costs. It may take us a couple of minutes at the end to get everyone's payments processed, so if you can bear with us that would be much appreciated.
- Please sign the consent form when you are ready and hand it back to us.

Demographics

When you have signed the consent form, there is another piece of paper with 4 questions on it. Please answer these questions. You don't need to write your name anywhere on this piece of paper. All the answers you give are completely confidential and it will not be possible to identify you by your answers. When you have completed the questions you can turn over the sheet and hand it back to us.

Introductions [~10 min]

Befofe we get started, we'll start with some introductions. I'd like us to go around the room and if everyone can tell us: What's your name? Where are you from? What's one of your favorite apps?

I'll start…

Health apps [~15 min]
We're going to start talking about smartphone apps for health. If you haven't used health apps before that's fine. We are really happy to have a diverse mix of people with different experiences of apps.

There are lots of health related apps out there. Some popular ones include calorie tracking apps, activity trackers, apps to help with quitting smoking or alcohol. I know not all you have used them.

Positive
I want to better understand your experiences with health apps – can you tell us about a positive experience you've had with a health app? Was there an app you used that you liked?

Prompts
- *what did they do well?*
- *why you like them?*
  which ones you might tell other people about?

Negative
I also want to understand your negative experiences with health apps - can you tell us about a health app that you've used that you didn't like?

Prompts
- *where they've failed?*
- *what you haven't liked about them?*
- *which ones you delete from your phones?*

If there's anyone who hasn't used a health app, why not? What would appeal to you about health apps?

Mental health apps [~25 min]
There are also apps that relate more specifically to mental health. What kind of app do you think of when you think of a mental health app?

Prompts
- *If there was a whole category of mental health apps, what kinds of apps would be there?*
  - How do you know an app helps mental health?

Why would you (or someone you know) use a mental health app?

Prompts
- *These may be apps that relate to stress, anxiety, depression, post-traumatic stress disorder, or other mental health problems.*
- *Popular mental health apps also include meditation or mindfulness apps.*

If you have used a mental health app before…
- What mental health apps do you like?
- What do you like about them?
- Are there apps you don't like? What don't you like about them?

If you were going to look for a mental health app…

- What would you want?
- Why would you want that?
- How would you know that it had it?
- Where would you find this information?
- What type of information would be useful?
- *What would be important factors to consider in your choice of app?* What helps you decide to download or not download an app?

PsyberGuide [~40 min]

Imagine there were a resource where all the mental health apps were collected. It would be kind of like the app store but ONLY for mental health apps.

- Does this sound like a useful resource?
- What do you like about this idea?
- What do you not like about this idea?
- What would make it useful?

Some resources like this actually exist. One is called PsyberGuide. It provides reviews of mental health apps.

We're going to go through PsyberGuide on the projector here and discuss it.

Because sometimes it's easier to react to something that exists, we're going to use PsyberGuide as an example, but feel free to use your imagination to tell us about things that don't exist too, or ideas you might have other than what we will see on this site.

Thinking just about the aesthetics or look of the website...

- What's your initial reaction to it?
- What do you like about it?
- What do you not like about it?
- Do you think it looks it easy to navigate?
- What do you think about the color scheme?
- So here we are on the homepage, what would you do next?
    - Where would you click next?

[walk through other pages of site, locating an app and reading through review - Headspace]

Thinking about the content...

- Do you find it easy to read and understand?
    - Do you think this (example) review gives you enough information to decide whether or not to use the app?
    - We include information on PsyberGuide about the research backing some of the apps. How much do you care about this information/how important is it?
    - If it is important, why?
    - If it's not important, why not?

What other information would you like to see on a website like PsyberGuide?
